# Effect of Dispersant on Disperse Dyeing in Silicone Waterless Dyeing System

**DOI:** 10.3390/polym15041046

**Published:** 2023-02-20

**Authors:** Jingru Chen, Liujun Pei, Wenhua Shi, Jingyuan Yi, Jiping Wang

**Affiliations:** 1 Engineering Research Center of Textile Chemistry and Clean Production, Shanghai University of Engineering Science, Shanghai 201620, China; 2 School of Textiles and Fashion, Shanghai University of Engineering Science, Shanghai 201620, China

**Keywords:** dispersant, hydrophilicity, disperse dye, polyester, silicone solvent

## Abstract

Traditional water-based dyeing of polyester textiles usually generates burdensome processes and a great deal of wastewater, which can no longer meet the green and sustainable developments in the textile dyeing industry. In the silicone waterless dyeing system, polyester textiles can be dyed with disperse dye without water. However, the dyeing performance of polyester textiles is influenced by the dispersant. In this study, the relationship between the properties of dispersants and disperse dyeing performance was studied. When the amount of dispersant NNO (2-Naphthalenesulfonic acid) was 1.2%, the exhaustion of disperse red 177 and the final K/S value of the dyed fabric improved to 94.18% and 14.73, respectively. However, the exhaustion of disperse red 177 was reduced from 90.73% to 82.61%, and the final K/S value of the dyed fabric was decreased from 14.77 to 14.01 when the dosage of MF (Naphthalenesulfonic acid) was 1.2%. Compared with different dyeing systems, the final uptake of disperse red 177 was 93.81% and 94.18% in traditional water-based and silicone waterless dyeing systems and the K/S value of the dyed fabric was almost the same. The washing and rubbing fastness (wet and dry) of the dyed fabric were found to be at a level of 4 or 4–5, and the light fastness of the dyed fabric was 3–4. If only the dispersant was added in the silicone waterless dyeing system, there was no leveling problems on dyed samples. Moreover, the maximum absorption wavelength of disperse red 177 was not changed after adding the dispersant. With an increasing amount of dispersant NNO, the solubility of the dye in the silicone solvent decreased, but it increased with an increasing amount of dispersant MF. In the relationship between dye exhaustion and dye solubility in a silicone waterless dyeing system, the exhaustion of dye was linearly and inversely proportional to the dye solubility. A dispersant with better hydrophilicity can decrease the solubility of the dye in dyeing media, and the dyeing performance of dye is better. Compared with previous studies, the exhaustion of dye was consistent with the ClogP value (hydrophobic constant) of the dyeing accelerant. Therefore, a dispersant with high hydrophilicity can reduce the solubility of dye and improve the exhaustion of disperse dye in a silicone waterless dyeing system. Moreover, the color fastness of the dyed fabric did not change before and after adding the dispersant.

## 1. Introduction

Dyeing is an important part of the textile industry. Traditional disperse dyeing usually involve harsh conditions such as strongly dispersant solutions and a high dyeing temperature (above 100 °C) [1,2,3]. Moreover, a large amount of alkali and sodium hydrosulfite is used in the reduction cleaning process to reduce the loose dye, which is adsorbed on the fiber surface. Therefore, water-based disperse dyeing and the reduction cleaning process produce a large amount of wastewater, which is not environmentally friendly [4,5]. Disperse dyeing effluent contains many chemicals containing carcinogens and mutagen chemicals such as disperse dye, dispersant, detergent, salts, etc., and causes serious environmental problems [6,7]. As a result, disperse dyeing in aqueous solutions introduces problems such as the assumption of a large amount of water and hazardous industrial effluents, leading to large energy and environmental challenges [8,9,10,11,12]. Therefore, it is urgent and imperative to develop clean dyeing techniques to replace the traditional water-based dyeing method [13,14,15].

To achieve a green sustainable dyeing technology for disperse dye, researchers have investigated many dyeing technologies, particularly waterless dyeing [16,17,18]. First, the vacuum sublimation dyeing process involves dye gasification and ring dyeing technology. Water in the dyeing process is avoided. After dyeing, disperse dyes are recycled and reused in the state of a solid powder [19]. However, there are fewer applicable dyes, and this method still needs continuous development. However, it does have the potential for industrialization. Secondly, the slow-release performance of disperse dye microcapsules is used by the microcapsule disperse dye dyeing technology [20]. It is suitable for the conventional dyeing process and no additives are needed. It can realize dyeing technology without additives and washing [21], but its synthesis process is more difficult to control, and the preparation materials are expensive. However, with the development of technology, these difficulties will inevitably be overcome to achieve industrialization. The supercritical carbon dioxide dyeing technology could achieve better dyeing of polyester fabric [22,23]. Moreover, this dyeing technology has been industrialized, and some products have been commercialized in the market. However, supercritical carbon dioxide dyeing technology must be realized with a high-pressure dyeing machine [24,25]. Organic solvent dyeing technology is also employed to dye polyester fabric [26]. However, this dyeing technology is often carried out using hydrocarbon solvents, which are not environmentally friendly [27,28], such as hexane, cyclohexane, and n-heptane as the continuous phase medium.

Unfortunately, to date, there are very few dyeing technologies for disperse dyes available in practice with the applicability of a waterless dyeing system, although polyester fabrics are dyed using an environmentally friendly method [29,30,31,32]. Therefore, the key to waterless dyeing technology is the dyeing medium in the dyeing process [33]. The silicone waterless dyeing system containing micro-dissolving disperse dyes offers this possibility. The micro-dissolving disperse dye technique did not need to be dispersed by a dispersant and did not require a reduction cleaning process and, therefore, can decrease energy consumption [34]. In our previous investigations, Zhang et al. synthesized new disperse dyes and studied their dyeing performance in a low-pressure waterless dyeing system [35]. However, the reason that the dispersant cannot be used in the silicone waterless dyeing system and the mechanism between the dispersant and accelerant have not been investigated.

In this investigation, two different kinds of conventional dispersants with similar structures were used in a silicone solvent dyeing system, and their influence on the exhaustion of disperse dye in this silicone waterless dyeing system was studied. C.I. Disperse Red 177 was selected to evaluate the influence of the dispersant on the exhaustion of dye and the color depth of the dyed fabric in the same dyeing bath. Moreover, the influence of the dispersant on the solubility of the dye in a silicone waterless dyeing system was systematically studied. The relationship between dye exhaustion and the solubility of the dye in the silicone solvent dyeing system was investigated. Furthermore, the relationship between the CLogP values of chemicals and the dyeing performance of dyes was also investigated. 

## 2. Material and Methods

### 2.1. Materials

**Figure 1:**
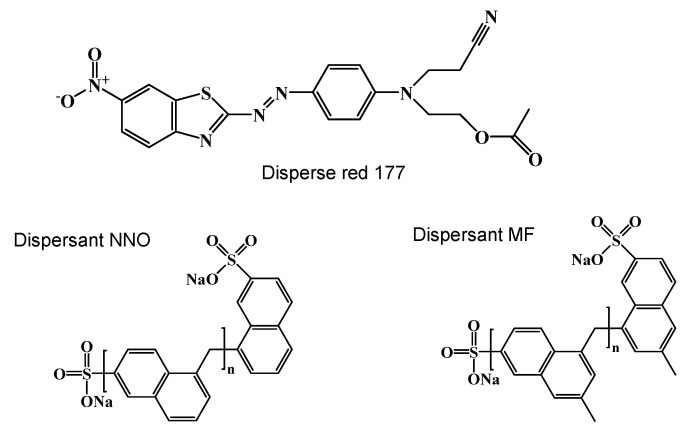
The molecular structure of disperse red 177, dispersant NNO, and MF.

Polyester fabric (150 D × 150 D) was provided by Suzhou Fengxiang Textile Technology Co., Ltd. (Suzhou, China). C.I. Disperse Red 177 (filter cake) was obtained from Zhejiang Longsheng Dye Chemical Co., Ltd. (Shaoxin, China). Dispersant NNO and dispersant MF (sodium salt of polynaphthalene sulphonic acid) were purchased from Anyang Shuanghuan Auxiliary Co., Ltd. (Anyang, China). The molecular structures of C.I. Disperse Red 177, dispersant NNO (2-Naphthalenesulfonic acid), and dispersant MF (Naphthalenesulfonic acid) are shown in Figure 1. Dimethyl sulfoxide (DMSO) was purchased from Shanghai Titan Technology Co., Ltd. (Shanghai, China). Gamma-butyrolactone (GBL) was purchased from Wuhan Huaxiang Kejie Biotechnology Co. Ltd. (Wuhan, China). Methanol (Meth) and ethyl alcohol (EtOH) were purchased from Shanghai Kaiin Chemical Co. Ltd. (Shanghai, China). The silicone solvent was supplied by GE Toshiba Silicone Ltd. (Jiujiang, China).

### 2.2. Dyeing Method

Dyeing was carried out on a dyeing machine (DYE-24, ShangHai Chain-Lih Automation Equipment Co., Ltd. Shanghai, China). Two grams of polyester fabric was dyed with 0.5% o.w.f (the weight amount of fabric) disperse dye at a liquor ratio of 15:1 in silicone solvent media. The amount of dispersant was 0%, 0.4%, 0.8%, 1.2%, 1.6%, and 2.0% o.w.f, respectively. Firstly, the dyeing temperature started at 25 ℃ and was increased to 80 °C at 6 °C /min, then it was increased to 140°C at 3 °C /min. The pressure of dyeing was 1.53 × 10^5^ Pa. After 60 min, the temperature was decreased to 80 °C at a rate of 6 °C/min, and then the dyed fabric was washed twice using the silicone solvent at a liquor ratio of 15:1 for 15 min.

Regarding water-based dyeing, 2 g of polyester fabric was dyed with 0.5% o.w.f disperse dye and 4 times the dispersant at a liquor ratio of 15:1 in a water solution at room temperature, and then the temperature was increased to 140 °C and dyeing was carried out for 60 min at 140 °C. The pressure of dyeing was 3.56 × 10^5^ Pa. After dyeing, reduction cleaning and neutralization cleaning were performed on the dyed fabric.

### 2.3. Exhaustion of Dye

The dye exhaustion refers to the proportion of dye that diffused into the PET fabric compared to the total dye. Firstly, the dye in the residue was extracted with DMSO, and then the samples were tested with an ultraviolet-visible spectrophotometer (UV-2600, Shimadzu Instrument Suzhou Co., Ltd. Suzhou, China). The maximum wavelength of disperse red 177 in DMSO is 520 nm. Each sample was tested 3 times, and its average value was employed for analysis. The exhaustion of disperse dye was calculated using Equation (1):

(1) $$E(\%)=\left(1-\frac{C_{1}V_{1}}{C_{0}V_{0}}\right)\times 100\%$$

where E (%) refers to the exhaustion of dye. C_0_ (g/L) is the dye concentration of the dyeing bath and C_1_ (g/L) is the dye concentration of the dyeing residual solution after dyeing. V_0_ (L) is the volume of the dyeing bath and V_1_ (L) is the volume of the residual dye solution after dyeing.

### 2.4. Color Depth of Dyed Fabric

The K/S value represents the color depth of the dyed sample, which was calculated by determining the reflectance R of the dyed fabric at the wavelength of minimum reflectance (maximum absorbance). The K/S value is calculated by the Kubelka–Munk formula (Equation (2)). Each sample was tested 3 times, and its average value was employed for analysis.

(2) $$\frac{K}{S}=\frac{{\left(1-R\right)}^{2}}{2R}$$

where K represents the absorption coefficient of the dyed fabric and S represents the scattering coefficient of the dyed fabric. R is the minimum reflectance of the dyed fabric.

### 2.5. Dyeing Level Property

Twelve points were randomly selected to test their K/S values, which were tested at the maximum absorption wavelength (λ_max_ = 520 nm). To characterize the level dyeing property of the dyed fabric, the CV (Coefficient of Variation) value was calculated by Equations (3) and (4). Each sample was tested 3 times and its average value was employed for analysis.

(3) $$S=\sqrt{\frac{\sum_{i=1}^{n}{\left({K/S}_{i}-\bar{K/S}\right)}^{2}}{n-1}}$$

(4) $$\mathrm{CV}\%=\frac{S}{\bar{K/S}}\times 100\%$$

### 2.6. Color Fastness Test

To evaluate the durability of the dyed fabric, the colorfastness to washing and rubbing of the dyed samples was measured according to ISO 105-C06 [36] and ISO 105-E04:2013 [37] testing standards, respectively. Colorfastness to washing was assessed with respect to the color change in samples and staining on the multifiber fabric. Rubbing fastness was evaluated in dry and wet conditions. According to ISO 105-B02 [38], the light fastness of the dyed fabric was also measured. 

### 2.7. Solubility of Disperse Dye

To test the saturation concentration of the dye in the silicone solvent, an excess amount of dye was placed in the silicone solvent at room temperature (25 °C), and then the temperature was increased to 140 °C and maintained for 60 min. Two milliliters of the dye solution at 140 °C were extracted with 20 mL of DMSO. The concentration of dye was determined via spectrophotometry, and the solubility of the dye was calculated by Equation (5). Each sample was tested 3 times, and its average value was employed for analysis.

(5) $$R=\frac{M}{V}$$

where R is the solubility (mg/L), M is the dissolved dye (mg), and V is the volume of the silicone solvent (L).

### 2.8. CLogP Value of Dye

To investigate the influence of the dispersant on the solubility of disperse dye in the silicone dyeing system, different dyeing accelerants, e.g., GBL, DMSO, MeTH, EtOH, and water, were selected, and their influence on disperse red 177 in the silicone dyeing system was investigated. The structures of dyeing accelerants were drawn using the chemical calculation software Chemdraw and their hydrophobic constant CLogP values were calculated. According to the dyeing method of Section 2.2, the polyester fabric was dyed with 20% (o.w.f) or without adding dyeing accelerant to investigate the influence of the dyeing accelerant on the dyeing property of disperse dye.

## 3. Results and Discussion

### 3.1. Exhaustion of Dye and Color Depth of Dyed Fabric

**Figure 2:**
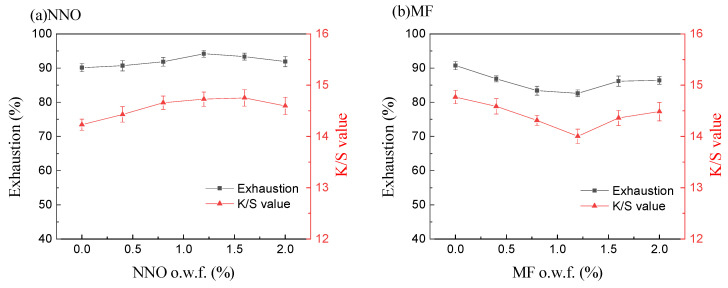
Influence of NNO (**a**) and MF (**b**) on the exhaustion of dye and the final K/S value of the dyed fabric.

Different amounts of dispersant were examined to determine the dye exhaustion and the final K/S value of the dyed fabric. The results of dye exhaustion and final K/S values are shown in Figure 2. As shown in Figure 2a, when there was no dispersant in the silicone waterless dyeing system, the exhaustion of disperse red 177 was 90.14, and the final K/S value of the dyed fabric was 14.26. With an increasing amount of dispersant NNO, the exhaustion of disperse red 177 and the final K/S value of the dyed fabric were increased. For example, when the amount of dispersant NNO was 1.2% o.w.f, the dye exhaustion and the final K/S value of the dyed fabric improved to 94.18% and 14.73, respectively. Afterward, as the amount of dispersant NNO continued to increase, the dye exhaustion decreased slightly, and the color depth of the dyed fabric decreased slightly too. After the amount of dye was confirmed, the influence of dispersant on disperse dyeing reached the maximum at 1.2% o.w.f, and continuing to increase its dosage did not generate any significant results.

Consequently, several homologous series of dispersant NNO as dispersant MF was further investigated as shown in Figure 2b. With the increasing amount of dispersant MF, both the dye exhaustion and the final K/S value of the dyed fabric were significantly decreased to a lower percentage. The disperse red 177 exhaustion was reduced from 90.73% to 82.61% with the addition of dispersant MF (1.2%, o.w.f), and the final K/S value of the dyed fabric decreased from 14.77 to 14.01 when the dosage of MF was 1.2% o.w.f. Afterward, as the amount of dispersant MF continued to increase, the dye exhaustion increased slightly, and the color depth of the dyed fabric increased slightly too. The reason might be that the dispersant affects the solubility of the dye in the silicone solvent dyeing system [39], and the aggregation of dye in the silicone solvent dyeing system might be influenced [40].

### 3.2. Dyeing Performance in Silicone Solvent Dyeing System and Traditional Water Base

Upon comparing different dyeing systems, the final uptake of disperse red 177 was 93.81% and 94.18% in the traditional water-based and silicone waterless dyeing systems, and the K/S value of the dyed fabric was almost the same. The washing colorfastness and rubbing of the dyed fabrics were tested in the silicone solvent dyeing system and traditional water-based system. It can be seen in Table 1 that the grayscale rating of the shade change and staining of adjacent fabrics (cotton, polyester, acrylic, wool, and acetate) were found to be very good (4–5 or 5) in the silicone solvent dyeing system and the traditional water-based system. Similarly, the dry and wet rubbing fastness of the dyed fabrics was found to be at a level of 4 or 4–5. 

Moreover, the application of the light fastness test method may be extended to include testing the color fastness to light of the dyed fabric in water and silicone solvent dyeing systems. After light exposure for a fixed period, the percentage changes in color between the exposed and unexposed parts of the blue wool fabrics and the measured samples were tested on a spectrophotometer and measured. From Table 1, the light fastness of the dyed fabric was 3–4, indicating that the silicone solvent had no influence on the light fastness of the dyed fabric. Therefore, disperse dyeing in the silicone solvent dyeing system does not affect the fastness properties of the polyester fabric. The product quality is fully compatible with international standards.

We investigated the influence of dispersant on the color fastness of the dyed fabric in the silicone solvent dyeing system. As shown in Table 2, if there was no dispersant in the silicone solvent dyeing system, the washing colorfastness and rubbing of dyed fabrics (control sample) were 4–5 or 5 and the light fastness was 3–4. However, the staining of the dyed fabric to cotton, acrylic, wool, and acetate was 5; that is, the staining fastness improved by a half level. Moreover, the rubbing fastness of the dyed fabric was also improved from 4–5 to 5. Before and after adding the dispersant, the light fastness did not change.

### 3.3. Dyeing Level Property of Dyed Fabrics

**Figure 3:**
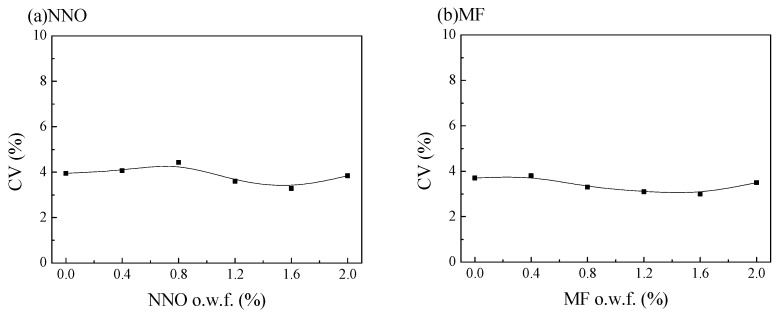
Influence of dispersant NNO (**a**) and MF (**b**) on the dyeing levelness (CV value) of the dyed fabric.

Figure 3 shows the influence of dispersant NNO and MF on the levelness of disperse dyed polyester fabrics in a low-pressure non-aqueous dyeing system. When there was no dispersant, the CV value of the dyed fabric was approximately 3.82. When the dosage of dispersant NNO and MF was 0.4%, 0.8%, 1.2%, 1.6%, and 2.0% o.w.f, the CV value of the dyed fabric was approximately 3.83 and 3.81, respectively. Therefore, different amounts of dispersant were used during polyester dyeing in the silicone solvent dyeing system, but the CV values of the dyed fabrics did not change much. Therefore, there were no leveling problems in fabrics when only the dispersant was added to the silicone waterless dyeing system.

**Figure 4:**
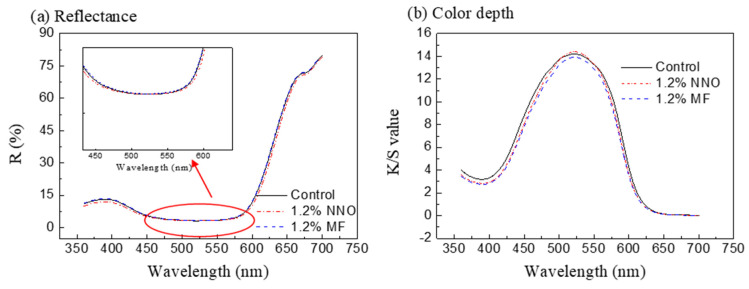
Influence of dispersant on the reflectance (**a**) and the color depth (**b**) of the dyed fabric.

In the silicone waterless dyeing system, the dispersant may influence the maximum absorption wavelength of disperse red 177. As shown in Figure 4a, the reflectance of the dyed fabric decreased with an increasing wavelength, and the minimum reflectance of the dyed fabric occurred at 520 nm in the silicone waterless dyeing system. From 520 nm to 700 nm, the reflectance of the dyed fabric increased. Compared with control samples (no dispersant), the minimum reflectance of the dyed fabric did not change after adding dispersant NNO or MF in the silicone waterless dyeing system.

From the results of Figure 4b, the maximum absorption wavelength of disperse red 177 was at 520 nm when there was no dispersant during dyeing. When dispersant NNO and MF were used in the silicone waterless dyeing system, the maximum absorption wavelength of disperse red 177 did not change. For the color depth, the K/S value of the dyed fabric increased from 14.26 to 14.73 when 1.2% o.w.f of NNO was employed during dyeing, but it decreased from 14.77 to 14.01 when the dosage of MF was 1.2% o.w.f. Therefore, the dispersant has no influence on the minimum reflectance and the maximum absorption wavelength of dyed fabric, rather it only influences the adsorption of dye on polyester fabric.

### 3.4. Solubility of Disperse Dye

It is well known that disperse dye can hardly dissolve in a water solution due to its hydrophobicity [41,42]. Meanwhile, the final uptake of disperse dye is determined by the hydrophobic interactions between hydrophobic dyes and hydrophobic fabrics [43]. The solubility of the disperse dye is inversely proportional to the final K/S value and the uptake of dye in the silicone solvent dyeing system.

**Figure 5:**
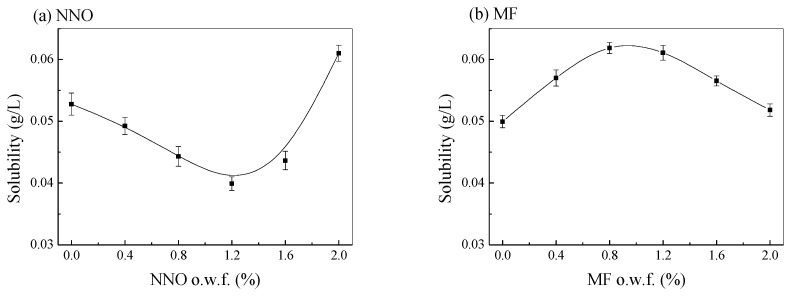
Influence of NNO (**a**) and MF (**b**) on the solubility of disperse red 177 in silicone waterless dyeing system.

As shown in Figure 5, different amounts of dispersants have a great effect on the solubility of disperse red 177 in the silicone solvent dyeing system. When there was no dispersant in the silicone solvent dyeing system, the solubility of disperse dye 177 was 0.052 g/L. With an increasing amount of dispersant NNO, the solubility of disperse red 177 decreased in the silicone waterless dyeing system. For example, the solubility of disperse red 177 in the silicone medium decreased from 0.052 g/L to 0.039 g/L under 1.2% o.w.f of dispersant NNO, which showed that the addition of dispersant NNO can reduce the dye solubility in the silicone dyeing medium. Afterward, as the amount of dispersant NNO continued to increase, the solubility of disperse red 177 increased slightly. The solubility of disperse red 177 in the silicone solvent was 0.061 g/L when the dosage of dispersant NNO was 2.0%, o.w.f. Compared with different dispersants, when the amount of dispersant MF increased from 0% to 0.8~1.2% o.w.f, the solubility of disperse red 177 in the silicone solvent increased from 0.052 g/L to 0.062 g/L. Afterward, as the amount of dispersant MF continued to increase, the solubility of disperse red 177 in the silicone solvent decreased slightly. For example, the solubility of dye decreased from 0.062 g/L to 0.053 g/L when the dosage of dispersant NNO increased from 1.2% o.w.f to 2.0 %, o.w.f. Combined with Figure 2, both dye exhaustion and the final K/S values of the dyed fabrics decreased with the increasing solubility of disperse dye.

From the above results, it can be concluded that disperse dye cannot be dissolved completely in the silicone solvent. In the silicone solvent dyeing system, some of the dye dissolved in the dyeing medium, and part of the dye was shown as aggregation [35]. After adding the dispersant, the aggregation of disperse dye was influenced; that is, the equilibrium between the molecular state and the aggregation state of disperse dye was changed by the dispersant. Furthermore, the dispersant influenced the uptake of the dye in the silicone solvent dyeing system.

**Figure 6:**
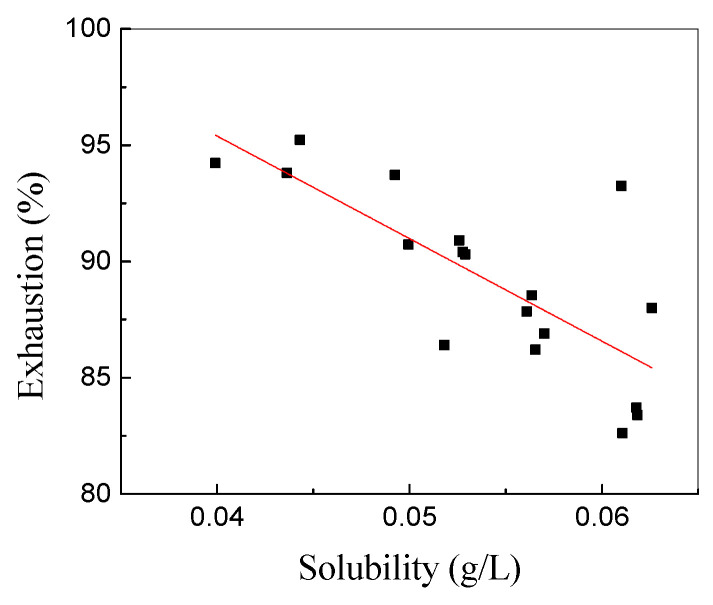
The relationship between the solubility of disperse dye and dye exhaustion.

To determine the relationship between dye exhaustion and dye solubility, the exhaustion of dye and dye solubility were plotted. As shown in Figure 6, disperse dye exhaustion on polyester fabric was inversely proportional to the solubility of dye in the silicone solvent dyeing system. The exhaustion of disperse red 177 was approximately 95% in the silicone solvent dyeing system when the solubility of the dye was 0.04 g/L. When the solubility of the dye increased from 0.04 g/L to 0.06 g/L, the exhaustion of disperse red 177 decreased from 95% to 83%. In the case of dye solubility, the addition of the dispersant can influence the solubility of the dye in the silicone solvent dyeing system, and the increase in solubility has a negative effect on the final dye uptake and the final K/S value of the dyed fabric. 

### 3.5. Relationship between Hydrophobic Constant and Exhaustion

The LogP value refers to the hydrophobic constant, that is, the logarithm value of the distribution coefficient of a chemical in the oil–water phase. The ClogP value represents the hydrophobic constant of an organic compound. The smaller the ClogP value, the better the hydrophilicity of the chemical [44,45,46,47]. To further study the properties of the dispersant and its effect on the dye solubility in the silicone waterless dyeing system, the ClogP values of dispersants were calculated. The ClogP values of dispersants NNO and MF were 2.613 and 3.611, respectively. Among these two dispersants, dispersant NNO had the lowest ClogP and it could partially improve the dye exhaustion, while dispersant MF had more lipophilicity due to its larger ClogP value. The solubility of the dye in the silicone solvent dyeing system increased due to the increasing intermolecular force between the dispersant and the disperse dye, decreasing the dye exhaustion on polyester fabric.

**Figure 7:**
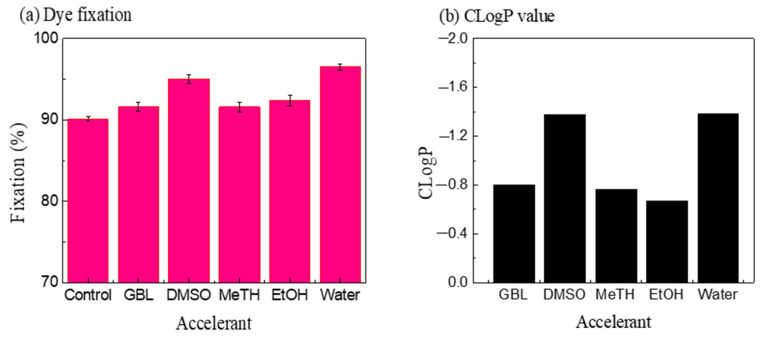
The relationship between the exhaustion of dye (**a**) and CLogP values of chemicals (**b**).

Figure 7 shows the dye uptake of C.I. Disperse Red 177 and the CLogP values of different chemicals added to the silicone solvent dyeing system. The relationship between the dye exhaustion rate and the CLogP value was essentially inversely proportional. The smaller the CLogP value, the more hydrophilic the chemical and the greater the final dye uptake rate. Because polyester fabric must be swollen, the small-molecule chemicals can interact with the dye molecules to make the dye easier to diffuse into the inner fiber, thereby increasing the exhaustion of dye in the silicone solvent dyeing system.

### 3.6. Dispersion Mechanism in Silicone Solvent Dyeing System

**Figure 8:**
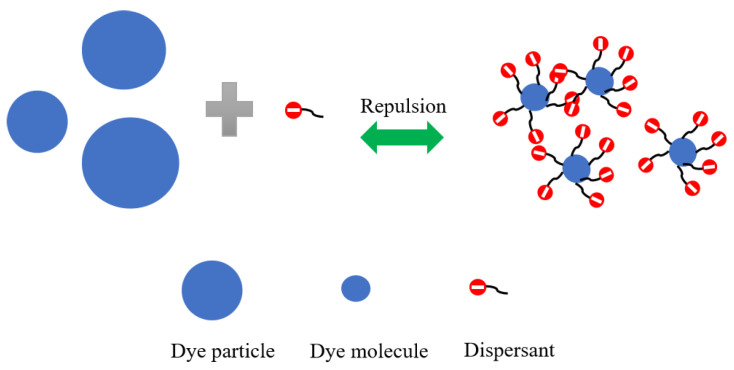
The dispersion mechanism of NNO/MF for disperse dye in silicone solvent dyeing system.

The dispersion mechanism of NNO/MF for disperse dye in the silicone low-pressure solvent dyeing system is shown in Figure 8. The volume of disperse dye is bigger than that of the dispersant, and these dispersants have certain negatively charged groups. The hydrophobic chain of the dispersant would adsorb on the surface of the dye. Because the hydrophilic chain is the carboxylic anion, its charge is a negative charge, which will result in the mutual exclusion of the negative charges between disperse dyes [48,49]. Therefore, there is a dispersion effect between disperse dyes. On the surface of the disperse dye, the naphthalene nucleus residue part of the dispersants is the most hydrophobic, primarily distributed in the innermost layer to be in contact with the dye. The carboxylic anion provides hydrophilicity [50,51], which caused the dispersant to be difficult to accumulate. The branch chain residue part grasped the dyed surface similar to a ‘‘bridge”, which established a finite adsorption interaction between the dispersant and disperse dye [52].

## 4. Conclusions

In conclusion, the final uptake of disperse red 177 was 93.81% and 94.18% in the traditional water-based system and the silicone waterless dyeing system, and the color depth of the dyed fabric was almost the same. The washing and rubbing fastness (wet and dry) of the dyed fabric were found at a level of 4 or 4–5, and the light fastness of the dyed fabric was 3–4. Dispersants NNO and MF, which are homologous dispersants, showed different effects on the polyester fabric dyeing in the silicone waterless dyeing system. Dispersant NNO has a positive effect on dye exhaustion, while dispersant MF can lead to a decrease in dye exhaustion during dyeing. When the amount of dispersant NNO was 1.2%, the dye exhaustion of the dye and the final K/S value of the dyed fabric were improved to 94.18% and 14.73, respectively. However, dye exhaustion was reduced from 90.73% to 82.61% and the final K/S value of the dyed fabric decreased from 14.77 to 14.01 when the dosage of MF was 1.2%. If only the dispersant was added to the silicone waterless dyeing system, there was no leveling problems on dyed samples. Moreover, the maximum absorption wavelength of disperse red 177 did not change after adding the dispersant. When dispersant NNO was employed in the silicone solvent dyeing system, the solubility of disperse red 177 in the silicone solvent decreased, which could improve the exhaustion of dye. Compared with dispersant NNO, dispersant MF can increase the solubility of the dye in the silicone solvent dyeing system, thus decreasing the exhaustion of dye. Furthermore, the hydrophobic constant values of dispersants were calculated and proved their influence on the dye solubility in the silicone solvent dyeing system. The smaller the ClogP values, the higher the dye exhaustion. From the investigation into dispersants’ effects on polyester fabric dyeing in a silicone solvent dyeing system, the dispersant with a better hydrophilic nature can decrease the dye solubility, which is beneficial to the final dye exhaustion.

## Figures and Tables

**Table 1:** Dyeing performance of polyester fabrics dyed in silicone waterless dyeing system.

Dyeing System	Dye Uptake(%)	K/S Value	Washing						Rubbing		Light
			Staining					Color Change	Dry	Wet	
			Cotton	Polyester	Acrylic	Wool	Acetate				
Water	93.8	14.51	4–5	4–5	5	4–5	5	4–5	4–5	4	3–4
Silicone	94.2	14.73	4–5	4–5	5	4–5	5	4–5	4–5	4	3–4

**Table 2:** Influence of dispersant on the dyed polyester fabrics dyed color fastness in silicone waterless dyeing system.

Dispersant	Washing						Rubbing		Light
	Staining					Color Change	Dry	Wet	
	Cotton	Polyester	Acrylic	Wool	Acetate				
Control	4–5	4–5	5	4–5	5	4–5	4–5	4	3–4
NNO	5	4–5	5	5	5	4–5	5	4–5	3–4
MF	5	4–5	5	5	5	4–5	5	4–5	3–4

## Data Availability

The data presented in this study are available on request from the corresponding author.
